# Enhanced Autophagy in Polycystic Kidneys of AQP11 Null Mice

**DOI:** 10.3390/ijms17121993

**Published:** 2016-11-30

**Authors:** Yasuko Tanaka, Mayumi Watari, Tatsuya Saito, Yoshiyuki Morishita, Kenichi Ishibashi

**Affiliations:** 1 Department of Pathophysiology, Faculty of Pharmacy, Meiji Pharmaceutical University, 2-522-1 Noshio, Kiyose, Tokyo 204-8588, Japan; mayuwata1204@icloud.com (M.W.); tsaito@my-pharm.ac.jp (T.S.); kishiba@my-pharm.ac.jp (K.I.); 2 Department of Nephrology, Saitama Medical Center, Jichi Medical University, 1-847 Ohmiya, Saitama-City, Saitama 330-8503, Japan; ymori@jichi.ac.jp

**Keywords:** proximal tubule, LC3, ER stress, apoptosis, PKD

## Abstract

Aquaporin-11 (AQP11) is an intracellular water channel expressed at the endoplasmic reticulum (ER) of the proximal tubule. Its gene disruption in mice leads to intracellular vacuole formation at one week and the subsequent development of polycystic kidneys by three weeks. As the damaged proximal tubular cells with intracellular vacuoles form cysts later, we postulated that autophagy may play a role in the cyst formation and examined autophagy activity before and after cyst development in AQP11(−/−) kidneys. PCR analysis showed the increased expression of the transcript encoding LC3 (Map1lc3b) as well as other autophagy-related genes in AQP11(−/−) mice. Using green fluorescent protein (GFP)-LC3 transgenic mice and AQP11(−/−) mice, we found that the number of GFP-LC3–positive puncta was increased in the proximal tubule of AQP11(−/−) mice before the cyst formation. Interestingly, they were also observed in the cyst-lining epithelial cell. Further PCR analyses revealed the enhanced expression of apoptosis-related and ER stress–related caspase genes before and after the cyst formation, which may cause the enhanced autophagy. These results suggest the involvement of autophagy in the development and maintenance of kidney cysts in AQP11(−/−) mice.

## 1. Introduction

Aquaporin-11 (AQP11) is a member of the aquaporin family that is expressed widely in mammalian tissues [1,2]. It is not expressed at the plasma membrane but uniquely at the membrane of intracellular organelles such as the endoplasmic reticulum (ER) [1,2], which makes its functional studies difficult and the results have been controversial. Some studies have reported water and glycerol transport [3,4,5], while others have failed to show any water transport activity [2]. Surprisingly, AQP11 gene disruption in mice showed intracellular vacuole formation in the proximal tubule at one week after birth, suggesting its important role in kidney development and function [1]. As AQP11 is mostly expressed in the ER of proximal tubular cells [1], AQP11 may play an important role in the ER and its disruption may lead to cyst formation. Very recently, the role of AQP11 in the glycosylation and trafficking of polycystin-1 (PC-1) from the ER to the plasma membrane has been reported [6]. As PC-1 is a gene product responsible for autosomal dominant polycystic kidney disease (ADPKD), cyst formation in AQP11-null (AQP11(−/−)) mice may be caused by defective PC-1 trafficking which is limited to the proximal tubule.

We previously reported the up-regulation of the genes involved in ER stress and apoptosis in the kidney of AQP11(−/−) mice [7]. Increased apoptosis has been observed in some animal models of polycystic kidneys as well [8,9,10], in which initial apoptosis is generally followed by increased cell proliferation and cyst formation. However, the causal relationship between apoptosis and cell proliferation is not clear. Interestingly, enhanced autophagy has also been observed in another polycystic kidney disease model [11]. We then postulated that autophagy may facilitate cell survival but not sufficiently, thus leading to an aberrant cell proliferation with eventual cyst formation.

Autophagy is a general term used for describing the degradation of cytoplasmic components within lysosomes [12], which will be important for cell survival by clearing damaged proteins and organelles from the cytoplasm and recycling their contents via a lysosomal pathway. In the kidney, autophagy has been shown to be an important mechanism for cellular homeostasis and survival during stressed pathologic conditions, such as ischemia-reperfusion injury and cisplatin cytotoxicity [13,14,15]. Thus, the induction of autophagy will be necessary for the damaged vacuolated cells in the AQP11(−/−) kidney to survive even though they are transformed to cyst epithelia of polycystic kidneys.

Three types of autophagy (macroautophagy, microautophagy, and chaperone-mediated autophagy) have been identified [12]. The most widely examined one is macroautophagy which is mediated by a unique double-membraned organelle, the autophagosome [12]. The activity of macroautophagy is monitored by the expression of LC3 (microtubule-associated protein light chain 3) specifically expressed at the autophagosomal inner membrane. Conveniently, green fluorescent protein (GFP)-LC3 has been employed as a marker for visualizing autophagy in vivo [16]. 

The purpose of this study was to examine the autophagy activity in the AQP11(−/−) kidney in the process of developing polycystic kidneys to document the role of autophagy in cell survival. To monitor autophagy in vivo, we introduced GFP-LC3 as a marker for autophagy in AQP11(−/−) mice and found enhanced autophagy activity throughout the development of the proximal tubule from vacuolated cells to cyst epithelia.

## 2. Results

### 2.1. Autophagy-Related Genes in the AQP11(−/−) Kidney

**Figure 1:**
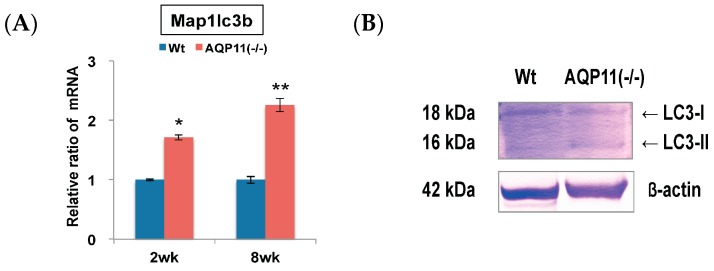
Quantitative reverse transcription polymerase chain reaction (qRT-PCR) analysis and Western blotting analysis of an autophagy-related gene and protein in the kidney. (**A**) The expression levels of Map1lc3b, an autophagy-related gene, were compared between the kidneys of AQP11(−/−) mice and wild-type mice at two weeks (wk) and at eight weeks. The expression level in the age-matched wild-type kidney is arbitrarily normalized to 1. The results are shown by the mean +/− SD. * *p* < 0.005 and ** *p* < 0.025 as compared with wild type (*n* = 5 per group in two-week-old mice, *n* = 3 per group at eight-week-old mice.); (**B**) The kidney expression of LC3 protein separated into LC3-I and LC3-II was compared between AQP11(−/−) mice and wild-type mice (Wt) at three weeks. The band of β-actin was used as an internal control. Similar observations were made in three separate sets of experiments.

The expression levels of autophagy-related genes in the kidney of AQP11(−/−) mice were compared with that of the wild type by qRT-PCR. The mRNA of microtubule-associated protein 1 light chain 3b (Map1lc3b) was examined, which is an isoform of LC3 attached to autophagosomes. We found up-regulation of Map1lc3b in AQP11(−/−) mice at the age of both two and eight weeks after birth (Figure 1A).

The protein levels of LC3 were further examined by Western blotting. As shown in Figure 1B, the band of LC3-I (18 kDa) was stronger than that of LC3-II (16 kDa) in wild-type mice, whereas the band of LC3-II was stronger than that of LC3-I in AQP11(−/−) mice. The level of LC3-II in AQP11(−/−) mice was 2.8 times stronger than that of wild-type mice (Supplemental Figure S1). It is known that during enhanced autophagy, LC3-II (active form) should be increased more than LC3-I (inactive form). Thus, the results indicated that autophagy was enhanced in the kidney of AQP11(−/−) mice.

**Figure 2:**
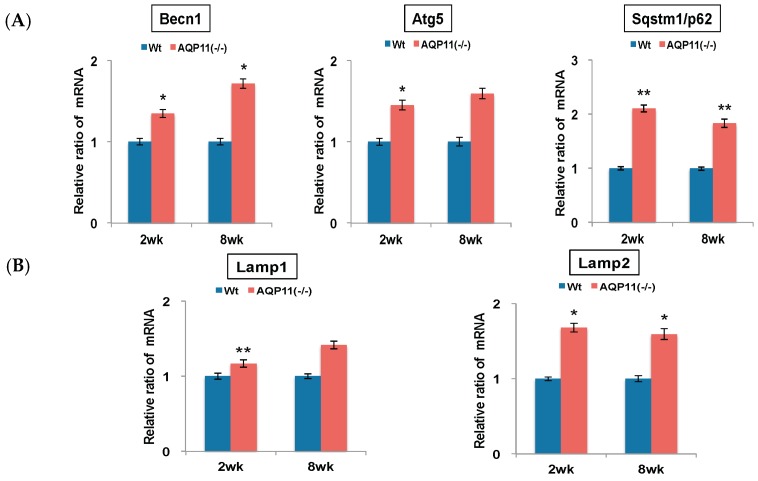
Quantitative analysis of autophagy-related genes in the kidney. All genes were compared by qRT-PCR between AQP11(−/−) mice and wild-type mice (Wt) at two weeks (wk) old and at eight weeks old. The expression level in the age-matched wild-type kidney is arbitrarily normalized to 1. Individual genes were examined as follows: (**A**) Becn1, Atg5, and Sqstm1/p62 as markers for early autophagosome; (**B**) Lamp1 and Lamp2 as markers for late autophagosome. The results are shown by the mean +/− SD. * *p* < 0.005 and ** *p* < 0.025 as compared with wild type (*n* = 4 to 5 per group at two weeks old, *n* = 3 per group at eight weeks old.).

To examine the enhanced autophagy activity further in AQP11(−/−) mice, the expression of other autophagy-related genes was examined. These genes are related to early autophagosome formation: Beclin1 (Becn1), autophagy related 5 (Atg5) and sequestosome 1 (Sqstm1/p62). The expression levels of Becn1 and Atg5 mRNA were up-regulated in the AQP11(−/−) kidney progressively from two to eight week old, while Sqstm1 was two-fold higher in the AQP11(−/−) kidney at the age of both two and eight weeks old (Figure 2A). Further separate analyses in the cortex and the medulla showed that the levels of these autophagy-related gene expressions were up-regulated in the cortex but not always in the medulla of AQP11(−/−) mice (Supplemental Figure S2). As the proximal tubule is dominant in the cortex, the results suggest the change in the whole kidney was caused by the change in the proximal tubule where AQP11 is selectively expressed [1].

As markers for late autophagosome formation, the expressions of lysosomal-associated membrane protein 1 (Lamp1) and lysosomal-associated membrane protein 2 (Lamp2) were examined. As shown in Figure 2B, all these genes were up-regulated in the AQP11(−/−) mice at the age of both two and eight weeks. Therefore, the increase of Map1lc3b may not be caused by the blocking of lysosomal fusions but most likely by the enhanced activity of autophagy. As the intracellular vacuoles are formed at the age of one to two weeks and subsequently the cysts develop at three to four weeks [1], the results indicated enhanced autophagy activity before and after the cyst formation in the kidney of AQP11(−/−) mice.

### 2.2. Autophagy Analysis in the AQP11(−/−) Kidney

**Figure 3:**
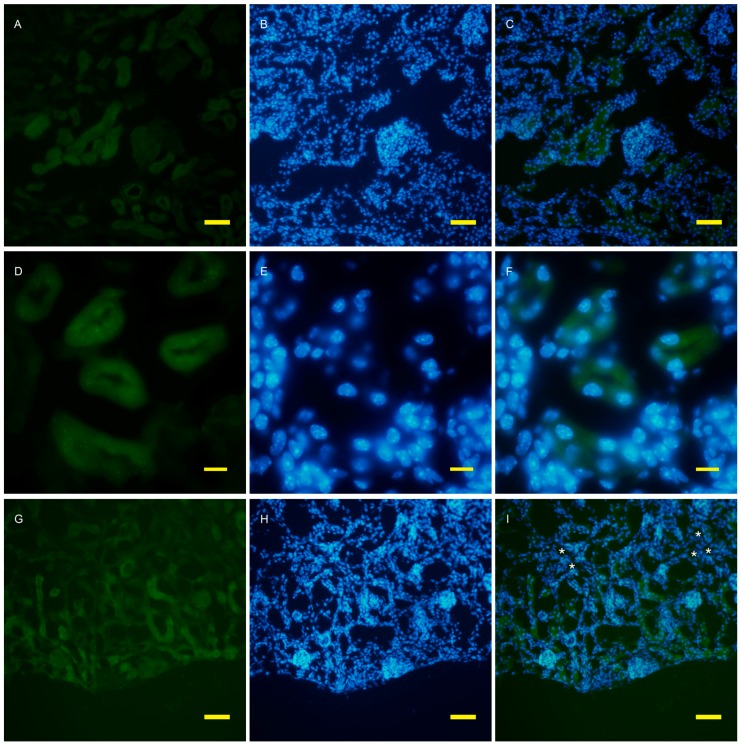

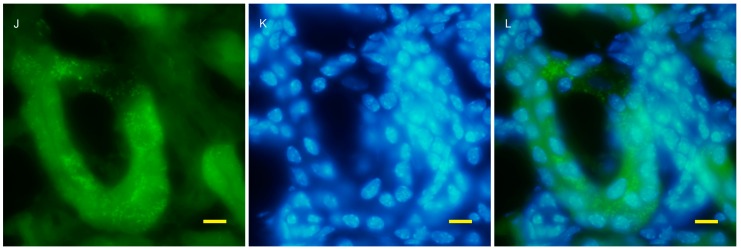
Fluorescent microscopy of the 2 week old kidney cortex of GFP-LC3 transgenic mice that were crossed with AQP11 knockout or wild-type mice. Green fluorescence shows GFP-LC3 (**A**,**D**,**G**,**J**), while blue fluorescence indicates nucleic staining with DAPI (**B**,**E**,**H**,**K**). Both images are merged (**C**,**F**,**I**,**L**); (**A**–**F**) are from wild-type mice and (**G**–**L**) are from AQP11(−/−) mice; (**A**–**C**) and (**G**–**H**) are at the lower magnification, while (**D**–**F**) and (**J**–**L**) are at the higher magnification, with bars indicating 50 and 10 μm length, respectively. The asterisks indicate the distal tubule (**I**). Similar observations were made in three separate sets of experiments.

**Figure 4:**
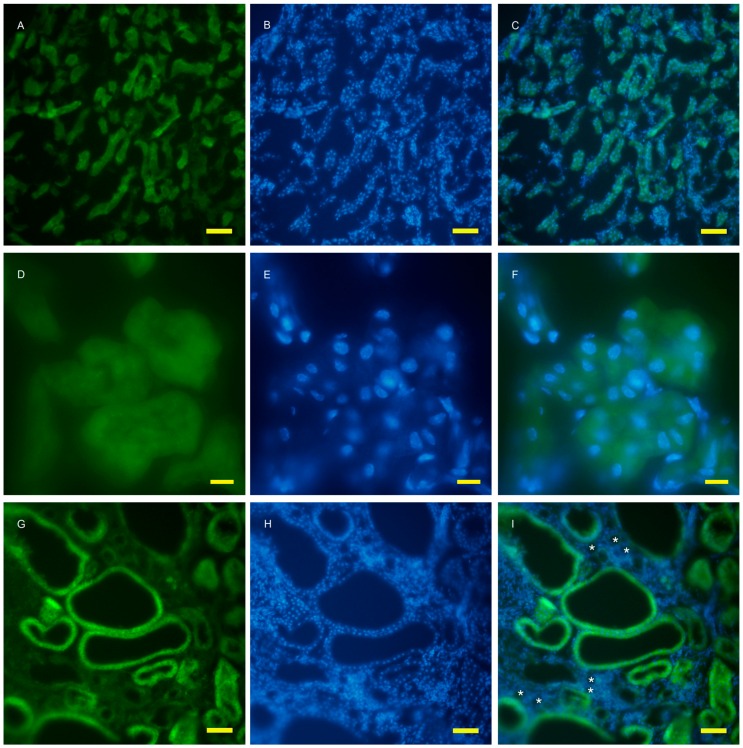

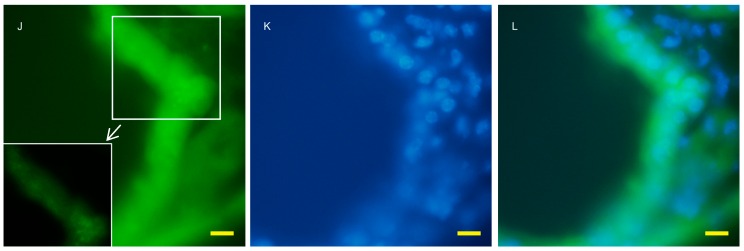
Fluorescent microscopy of the 7 week old kidney cortex of GFP-LC3 transgenic mice that were crossed with AQP11 knockout or wild-type mice. Green fluorescence shows GFP-LC3 (**A**,**D**,**G**,**J**), while blue fluorescence indicates nucleic staining (**B**,**E**,**H**,**K**). Both images are merged (**C**,**F**,**I**,**L**); (**A**–**F**) are wild-type and (**G**–**L**) are AQP11 −/−) mouse; (**A**–**C**) and (**G**–**H**) are at the lower magnification, while (**D**–**F**) and (**J**–**L**) are at the higher magnification. The asterisks indicate the distal tubule (**I**). The left lower frame of J showed enlarged puncta of GFP which were expressed in the proximal tubule. Bars indicate 50 and 10 μm length at each magnification. The asterisk indicates the distal tubule. Similar observations were made in three separate sets of experiments.

To examine autophagy at a cellular level, the amount of autophagosomes visualized by fluorescent puncta of GFP-LC3 was examined in wild and AQP11(−/−) kidneys [16,17]. At the age of two weeks, just before cyst formation, the GFP-LC3 signal was increased in the cortex (Figure 3G). At a higher magnification, GFP-LC3 tiny puncta were expressed on one surface and especially localized at proximal tubular cells, which were more intense in the AQP11(−/−) mice (Figure 3J) than in the wild type (Figure 3D). The difference was clearer with Image J analysis (Supplemental Figure S3). More GFP-LC3 puncta were observed at seven weeks old when cyst formation was completed (Figure 4G,J). Slight fluorescence in the wild type was most likely caused by nonspecific GFP expression as it was not in the form of puncta but in homogenous broader signals (Figure 4A,D). A distal tubule displayed minimum autophagy activities (indicated by asterisks in Figure 4I). Therefore, AQP11(−/−) mice had enhanced autophagy activity before and after cyst formation in agreement with the results of qRT-PCR (Figure 1A).

### 2.3. The Expression of Apoptosis-Related and ER Stress–Related Genes

**Figure 5:**
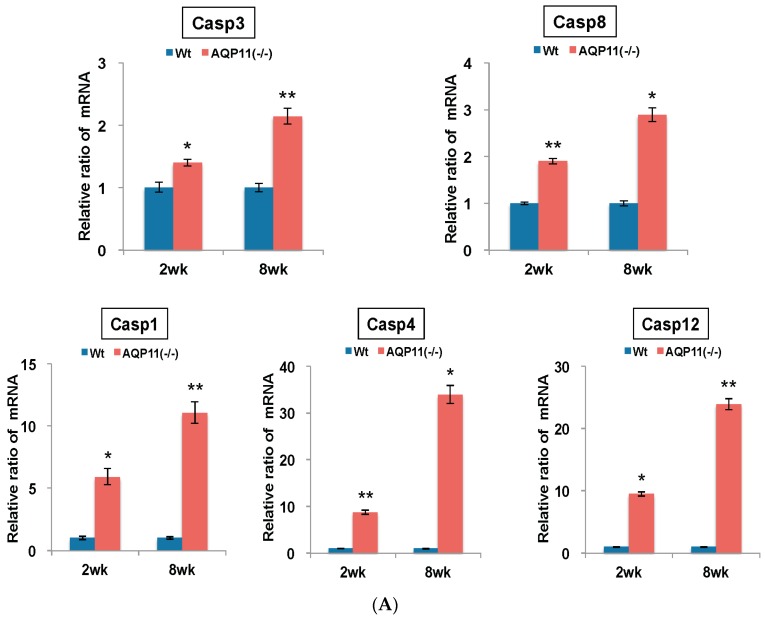

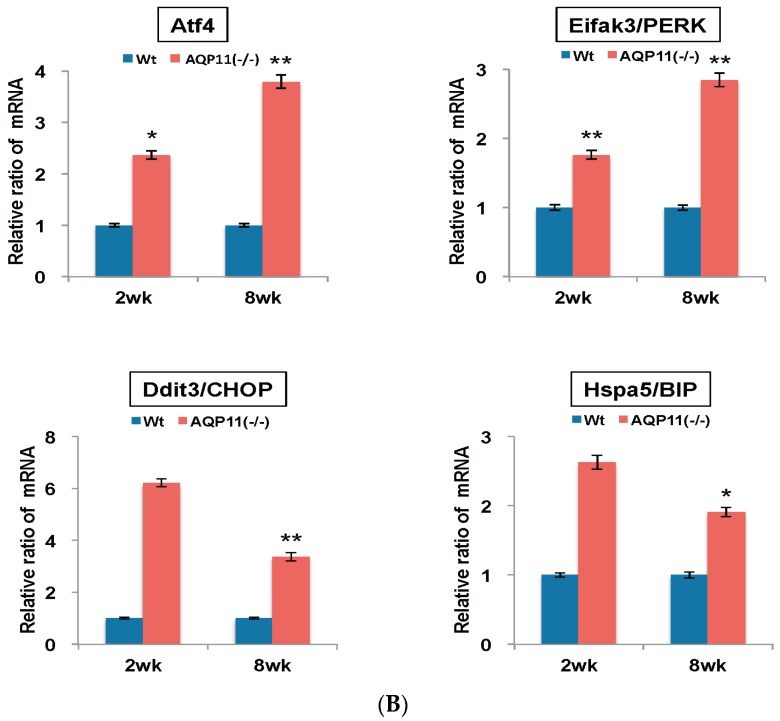
Quantitative analysis of mRNA expression in the kidney of AQP11(−/−) and wild-type mice at two and eight weeks old. All genes were compared by qRT-PCR between AQP11(−/−) mice and wild-type mice (Wt) at two weeks (wk) old and at eight weeks old. The expression levels in the age-matched wild-type kidney are arbitrarily normalized to one. (**A**) Expression of inflammation, apoptosis, and ER stress–related caspase family genes; (**B**) Expression of ER stress response genes related to Atf4/PERK signaling pathway. The results are shown by the mean +/− SD. The number of experiments was three to four each week. * *p* < 0.005 and ** *p* < 0.025 as compared with wild type (*n* = 4 to 5 per group at two weeks old, *n* = 3 per group at eight weeks old).

To examine the possible cause of the enhanced autophagy activity in the AQP11(−/−) kidney, the expression of caspase genes related to apoptosis, inflammation and ER stress was examined, which will induce autophagy. As indicators for the effector and the initiator of apoptosis, caspase 3 (Casp3) and caspase 8 (Casp8) were examined, respectively. As indicators for active inflammation, caspase 1 (Casp1) and caspase 4 (Casp4) were examined. As an indicator for ER stress, caspase12 (Casp12) was examined. The mRNA level of each caspase change in the kidney was quantified by qRT-PCR. As shown in Figure 5A, the mRNA expression levels of Casp3 and Casp8 were enhanced significantly at the age of two weeks and further at eight weeks in the AQP11(−/−) kidney, suggesting an augmented apoptotic activity. The mRNA expression level of Casp1 was also increased five-fold at the age of two weeks and 12-fold at eight weeks. Much higher expression levels were observed with Casp4 mRNA, by seven-fold at the age of two weeks and by 32-fold at eight weeks. Both results suggest the presence of an active inflammation in the AQP11(−/−) kidney. Finally, the mRNA expression levels of Casp12 was also enhanced nine-fold at the age of two weeks and 26-fold at eight weeks, suggesting the presence of ER stress. Thus, all the caspase genes examined were enhanced in the AQP11(−/−) kidney before (at two weeks) and after (at eight weeks) cyst formation with remarkably higher expressions of Casp1, Casp4 and Casp12. Taken together, our data suggest that the induction of autophagy in the AQP11(−/−) kidney was associated with apoptosis, inflammation, and ER stress. 

Next, the genes related to the apoptosis induced by ER stress were studied. Both activating transcription factor 4 (Atf4) and eukaryotic translation initiation factor 2 alpha (Eifak3) work in the PERK-Atf4 pathway and thereby lead to apoptosis through the integrated ER stress response. DNA-damage inducible transcript 3 (Ddit3/CHOP) is another downstream target of the PERK-Atf4 pathway. The expression of heat shock protein 5 (Hspa5/Bip) which is activated by accumulated altered proteins in the ER was also examined. As shown in Figure 5B, the mRNA expression levels of Atf4 and Eifak3 were two-fold higher at the age of two weeks and three-fold higher at eight weeks. Similarly, Ddit3 was six-fold higher at the age of two weeks and three-fold higher at eight weeks, and Hspa5 was 2.6-fold higher at the age of two weeks and two-fold higher at eight weeks (Figure 5B). The results further confirmed the enhanced ER stress activity before and after cyst formation, in agreement with the results of the caspase expression (Figure 5A). Our results partly agreed with the previous microarray data [7].

## 3. Discussion

Here we reported that autophagy was enhanced in the kidney of AQP11(−/−) mice before and even after cyst formation. The autophagy was most likely induced by ER stress with misprocessed proteins, as suggested by the report that AQP11 expressed at the ER is important for PC-1 trafficking to the plasma membrane with abnormal glycosylation [6]. As the proximal tubule suffers from extensive cell damage with intracellular vacuoles in the absence of AQP11 before cyst formation, higher autophagy activities will be necessary for the survival of these cells as previously reported in other kidney injury models [13,14,15]. Apoptosis may also be induced by ER stress to eliminate irreversibly damaged and dead cells from the tubule to support the recovery of the proximal tubule [7]. The relationship between autophagy and apoptosis is intriguing and worthy of further studies. 

It was unexpected to find a continued enhanced autophagy activity even after cyst formation where active cell damage seems to be absent. In fact, our quantitative gene expression studies of several caspases revealed much higher expression of apoptosis- and ER stress–related genes after cyst formation. The results suggest that autophagy will be important for the survival of cyst epithelia, as was the case with vacuolated cells. It is possible that the cyst epithelium may also suffer from a cellular damage in the absence of AQP11 with diminished glomerular filtration and narrowed vessels compressed by cysts, which eventually compromise the blood supply to cyst epithelia. As the progressive nature of renal failure in AQP11(−/−) mice suggests, the cyst epithelium may still need AQP11 and enhanced autophagy will be helpful for its survival. In fact, an enhanced autophagy activity has also been reported in other polycystic kidney disease (PKD) models [11,13]. However, cyst epithelia undergoing autophagy may be at the initiation phase and the surviving phase since both occur simultaneously and at different segments of the nephron in most PKD models. However, acute and selective damage to the proximal tubule in our model will make this distinction much easier [1]. Alternatively, autophagy could have been induced by ischemia produced by physical damage to blood vessels by expanded and cystic epithelia, which also induced ER stress and apoptosis. Further studies on the cause-effect relationship of autophagy and ER stress will need to be clarified.

The cyst formation in our model will be initiated by a defective ER function since abnormal glycosylation of polycystin-1 (PC-1) has been shown to cause its defective transfer to the plasma membrane [6]. We speculated that ER stress induced by the absence of AQP11 may trigger apoptosis in the proximal tubule while some cells may survive by simultaneously induced autophagy to form cysts with a defective PC-1 function. Interestingly, the cyst epithelium still seems to need autophagy for its survival. More studies on the relationship between the defective trafficking of PC-1 and autophagy will be necessary to clarify the role of autophagy in cyst formation and its survival. Further study will also be necessary to clarify the cause-effect relationship between autophagy, apoptosis and ER stress. If autophagy is be important for the survival of cysts, modulations of autophagy will be a novel therapy against the progression of PKD. 

We conclude that autophagy was induced in the proximal tubule of AQP11(−/−) mice associated with enhanced apoptosis and ER stress, and autophagy may be important for the development of cysts.

## 4. Materials and Methods

### 4.1. Mice

AQP11(−/−) mice were generated in our laboratory as previously reported [1]. GFP-LC3 heterozygous transgenic mice were obtained from RIKEN Bio-Resource Center in Japan [16]. Heterozygous AQP11 knockout mice (AQP11(+/−)) were interbred with heterozygous GFP-LC3 transgenic mice to generate GFP-LC3 transgenic mice in the background of AQP11(−/−). The genotyping for both AQP11 and GFP-LC3 were determined by PCR as previously reported [1,16].

### 4.2. Quantitative Real-Time PCR

Mice were anesthetized with pentobarbital and the whole body was perfused via the left ventricle of the heart with diethyl pyrocarbonate (DEPC, Nacalai Tesque, Kyoto, Japan) treated PBS (TaKaKa, Shiga, Japan). Kidneys were then harvested and immersed in RNA later (QIAGEN, Tokyo, Japan) until use. Total RNA was isolated using RNeasy Mini Kit (QIAGEN) and the aliquots of 50 ng were reverse transcribed with random primers according to the manufacturers’ instructions. Then, quantitative RT-PCR (qRT-PCR) was performed on the cDNA in triplets by Light Cycler Nano (Roche, Tokyo, Japan) with Fast Start Essential DNA Green Master (Roche) following the manufacturer’s instructions. The primer sets used in this study were summarized in Table 1. The relative amount of mRNA was calculated using GAPDH mRNA as an internal control with second Derivative Maximum method. A relative ratio was calculated by the gene expression of AQP11(−/−) mice relative to wild-type mice.

### 4.3. Western Blotting

After perfused with phosphate buffered saline (PBS), kidneys were isolated from the mice and then homogenized with a galas-Teflon homogenizer in Radio-Immunoprecipitation Assay (RIPA) buffer (Wako). After centrifugation at 10,000× *g* for 20 min at 4 °C, the supernatant was collected. The solubilized protein was added 4× SDS buffer (12% SDS, 25% Glycerol, 150 mM Tris-HCl (pH 7.0), 0.05% Bromophenol Blue and 6% β-mercaptoethanol) and equilibrated at room temperature for 30 min. The proteins (10 μg) were subjected to SDS-PAGE (15% gel, ATTO) and transferred to a Hybrond-P polyvinylidene difluoride membrane (GE Healthcare, Tokyo, Japan) by a semi-dry blotting apparatus. The membrane was blocked with 1% bovine serum albumin in TBST for 1 h at room temperature. The primary antibodies were rabbit anti-LC3 (MBL, PM036, 1:1000 dilution) and rabbit anti-β-actin (Abcam, ab25894, 1:10,000 dilution). The bands were visualized by ProtoBlot II AP System with Stabilized Substrate Kit (Promega, Tokyo, Japan, W3960). 

### 4.4. Fluorescent Microscopy

Mice were perfused as above with 4% paraformaldehyde (PFA, Sigma-Aldrich, Osaka, Japan) in PBS. Kidneys were then harvested and fixed in 4% PFA overnight. The fixed tissues were immersed in PBS containing 10%, 15% and 20% (*w*/*v*) sucrose in succession. Finally, the samples were embedded in OTC compound (Tissue-Tek, Sakura Finetek, Tokyo, Japan) and frozen at −30 °C until use. Cryosections were prepared in 8 μm thickness with the Leica CM1510S cryostat (LEICA). Nuclei were stained with 4′,6-diamidio-2-phenylindole (DAPI, Wako, Osaka, Japan). GFP and DAPI fluorescent signals were detected by an inverted fluorescent microscope (OLYMPUS, IX71) with respective filters.

### 4.5. Statistical Analysis

Comparisons between the two groups were performed using unpaired Student’s *t*-test. * *p* < 0.005 and ** *p* < 0.025 were considered to indicate a statistically significant difference. All data are expressed as means +/− SD.

### 4.6. Digital Imaging

The comparison of density by Western blotting analysis and heat map analysis of image were used Image J software 1.46r which was developed by National Institutes of health, USA (available online: http://imagej.nig.gov/ij). The density of western blotting were calculated by the Plot of Scan analysis. The heat map analysis of fluorescence image were used Surface Plot analysis.

## Figures and Tables

**Table 1:** Sequences of primers for qRT-PCR.

Genes	Forward	Reverse
*Atf4*	5′-aitgatggcttggccagtg-3′	5′-ccattttctccaacatccaatc-3′
*Atg5*	5′-aagtctgtccttccgcagtc-3′	5′-tgaagaaagttatctgggtagctca-3′
*Becn1*	5′-aggatggtgtctctcgaagatt-3′	5′-gatcagagtgaagctattagcactttc-3′
*Casp1*	5′-cccactgctgatagggtgac-3′	5′-gcataggtacataagaatgaactgga-3′
*Casp3*	5′-gaggctgacttcctgtatgctt-3′	5′-aaccacgacccgtccttt-3′
*Casp4*	5′-tgtcatctctttgatatattcctgaag-3′	5′-caaggttgcccgatcaat-3′
*Casp8*	5′-ttgaacaatgagatccccaaa-3′	5′-ccatttctacaaaaatttcaagcag-3′
*Casp12*	5′-gggaattagcacaggcaact-3′	5′-ttcttttcttctcagctacagcaa-3′
*Ddit3/CHOP*	5′-gcgacagagccagaataaca-3′	5′-gatgcacttccttctggaaca-3′
*Eifak3/PERK*	5′-ccttggtttcatctagcctca-3′	5′-atccagggaggggatgat-3′
*GAPDH*	5′-tgtccgtcgtggatctgac-3′	5′-cctgcttcaccaccttcttg-3′
*Hspa5/BIP*	5′-ctgaggcgtatttgggaaag-3′	5′-tcatgacattcagtccagcaa-3′
*Lamp1*	5′-cctacgagactgcgaatggt-3′	5′-ccacaagaactgccatttttc-3′
*Lamp2*	5′-aaggtgcaaccttttaatgtgac-3′	5′-tgtcatcatccagcgaacac-3′
*Map11c3b*	5′-ccccaccaagatcccagt-3′	5′-cgctcatgttcacgtggt-3′
*Sqstm1/p62*	5′-agacccctcacaggaaggac-3′	5′-catctgggagagggactcaa-3′

## Supplementary Materials

Supplementary materials can be found at www.mdpi.com/1422-0067/17/12/1993/s1.

## References

[1] Morishita Y., Matsuzaki T., Hara-chikuma M., Andoo A., Shimono M., Matsuki A., Kobayashi K., Ikeda M., Yamamoto T., Verkman A. (2005). Disruption of aquaporin-11 produces polycystic kidneys following vacuolization of the proximal tubule. Mol. Cell. Biol..

[2] Gorelick D.A., Praetorius J., Tsunenari T., Nielsen S., Agre P. (2006). Aquaporin-11: A channel protein lacking apparent transport function expressed in brain. BMC Biochem..

[3] Yakata K., Hiroaki Y., Ishibashi K., Sohara E., Sasaki S., Mitsuoka K., Fujiyoshi Y. (2007). Aquaporin-11 containing a divergent NPA motif has normal water channel activity. Biochim. Biophys. Acta.

[4] Yakata K., Tani K., Fujiyoshi Y. (2011). Water permeability and characterization of aquaporin. J. Struct. Biol..

[5] Madeira A., Fernández-Veledo S., Camps M., Zorzano A., Moura T.F., Ceperuelo Mallafré V., Vendrell J., Soveral G. (2014). Human aquaporin-11 is a water and glycerol channel and localizes in the vicinity of lipid droplets in human adipocytes. Obesity.

[6] Inoue Y., Sohara E., Kobayashi K., Chiga M., Rai T., Ishibashi K., Horie S., Su X., Zhou J., Sasaki S. (2014). Aberrant glycosylation and localization of polycystin-1 cause polycystic kidney in an AQP11 knockout model. J. Am. Soc. Nephrol..

[7] Okada S., Misaka T., Tanaka Y., Matsumoto I., Ishibashi K., Sasaki S., Abe K. (2008). Aquaporin-11 knockout mice and polycystic kidney disease animals share a common mechanism of cyst formation. FASEB J..

[8] Moser M., Pscherer A., Roth C., Becker J., Mücher G., Zerres K., Dixkens C., Weis J., Guay-Woodford L., Buettner R. (1997). Enhanced apoptotic cell death of renal epithelial cells in mice lacking transcription factor AP-2beta. Genes Dev..

[9] Trudel M., D’Agati V., Costantini F. (1991). C-myc as an inducer of polycystic kidney disease in transgenic mice. Kidney Int..

[10] Veis D.J., Sorenson C.M., Shutter J.R., Korsmeyer S.J. (1993). Bcl-2-deficient mice demonstrate fulminant lymphoid apoptosis, polycystic kidneys, and hypopigmented hair. Cell.

[11] Belibi F., Zafar I., Ravichandran K., Segvic A.B., Jani A., Ljubanovic D.G., Edelstein C.L. (2011). Hypoxia-inducible factor-1α (HIF-1α) and autophagy in polycystic kidney disease (PKD). Am. J. Physiol. Ren. Physiol..

[12] Maiuri M.C., Zalckvar E., Kimchi A., Kroemer G. (2007). Self-eating and self-killing: Crosstalk between autophagy and apoptosis. Nat. Rev. Mol. Cell Biol..

[13] Huber T.B., Edelstein C.L., Hartleben B., Inoki K., Jiang M., Koya D., Kume S., Lieberthal W., Pallet N., Quiroga A. (2012). Emerging role of autophagy in kidney function, diseases and aging. Autophagy.

[14] Periyasamy-Thandavan S., Jiang M., Wei Q., Smith R., Yin X.M., Dong Z. (2008). Autophagy is cytoprotective during cisplatin injury of renal proximal tubular cells. Kidney Int..

[15] Jiang M., Wei Q., Dong G., Komatsu M., Su Y., Dong Z. (2012). Autophagy in proximal tubules protects against acute kidney injury. Kidney Int..

[16] Mizushima N., Yamamoto A., Matsui M., Yoshimori T., Ohsumi Y. (2004). In vivo analysis of autophagy in response to nutrient starvation using transgenic mice expressing a fluorescent autophagosome marker. Mol. Biol. Cell.

[17] Kuma A., Hatano M., Matsui M., Yamamoto A., Nakaya H., Yoshimori T., Ohsumi Y., Tokuhisa T., Mizushima N. (2004). The role of autophagy during the early neonatal starvation period. Nature.

