# Supplementary material for: Enhanced Autophagy in Polycystic Kidneys of AQP11 Null Mice
Source: Int J Mol Sci. 2016 Nov 30;17(12):1993. doi: 10.3390/ijms17121993 (PMC5187793; doi:10.3390/ijms17121993)
Supplement: Supplementary file 1 [file ijms-17-01993-s001.pdf]

# Supplementary Materials: Enhanced Autophagy in Polycystic Kidneys of AQP11 Null Mice

**Yasuko Tanaka, Mayumi Watari, Tatsuya Saito, Yoshiyuki Morishita and Kenichi Ishibashi**

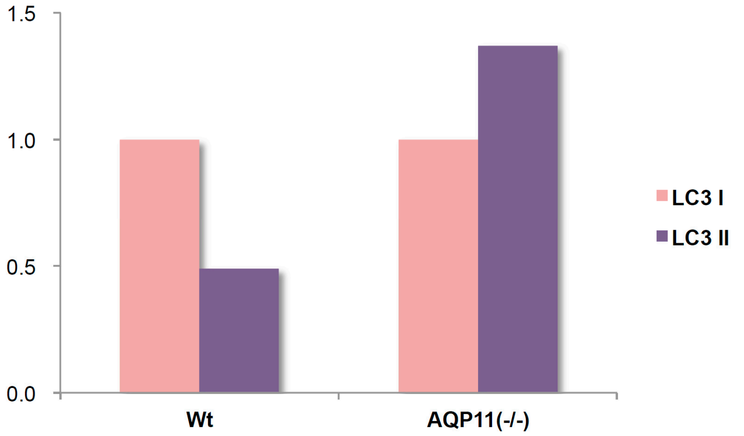


**Figure S1.** The western blo-ng of Figure 1B was analyzed by graphical method (ImageJ). It was showed that inac@ve form LC3 I of wild mice was in the standard (=1). The inac@ve form of both mice was the same density. The ac@ve form of LC3 II was half density for the inac@ve form of LC3 I in wild mice. In contrast, the expression of LC3 II in AQP11(−/−) mice was increased to 2.8 @mes for the expression of LC3 I in wild mice. The results suggest that autophagy was enhanced in the kidney of AQP11 (−/−).

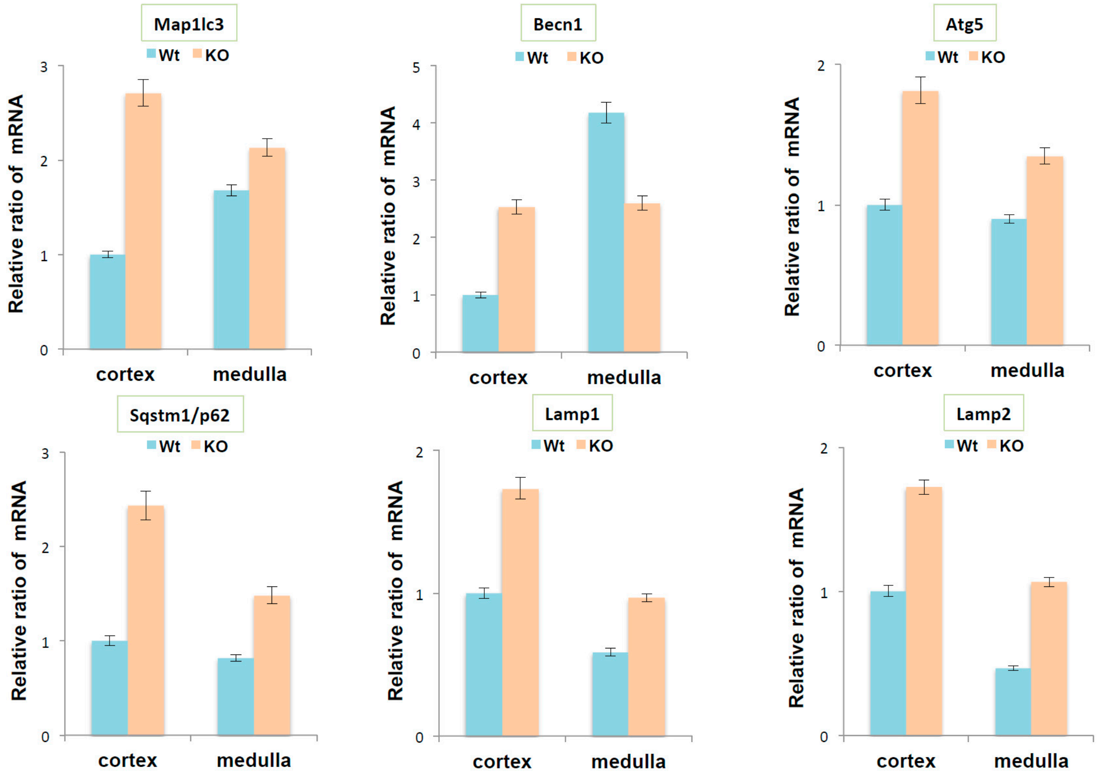


**Figure S2.** Quan@ta@ve analysis (qRT-PCR) for Map1lc3b (an autophagy marker), Becn1, Atg5 and Sqstm1/p62 (early augophagosome markers), and Lamp1 and Lamp2 (late autophagosome markers) in the kidney of 3 week old mice. The expression level of each gene was compared between AQP11(−/−) and wild type in the cortex and the medulla. The expression levels in the cortex of the wild type are arbitrarily normalized to one. The results are the mean +/− SE of three separate sets of experiments.

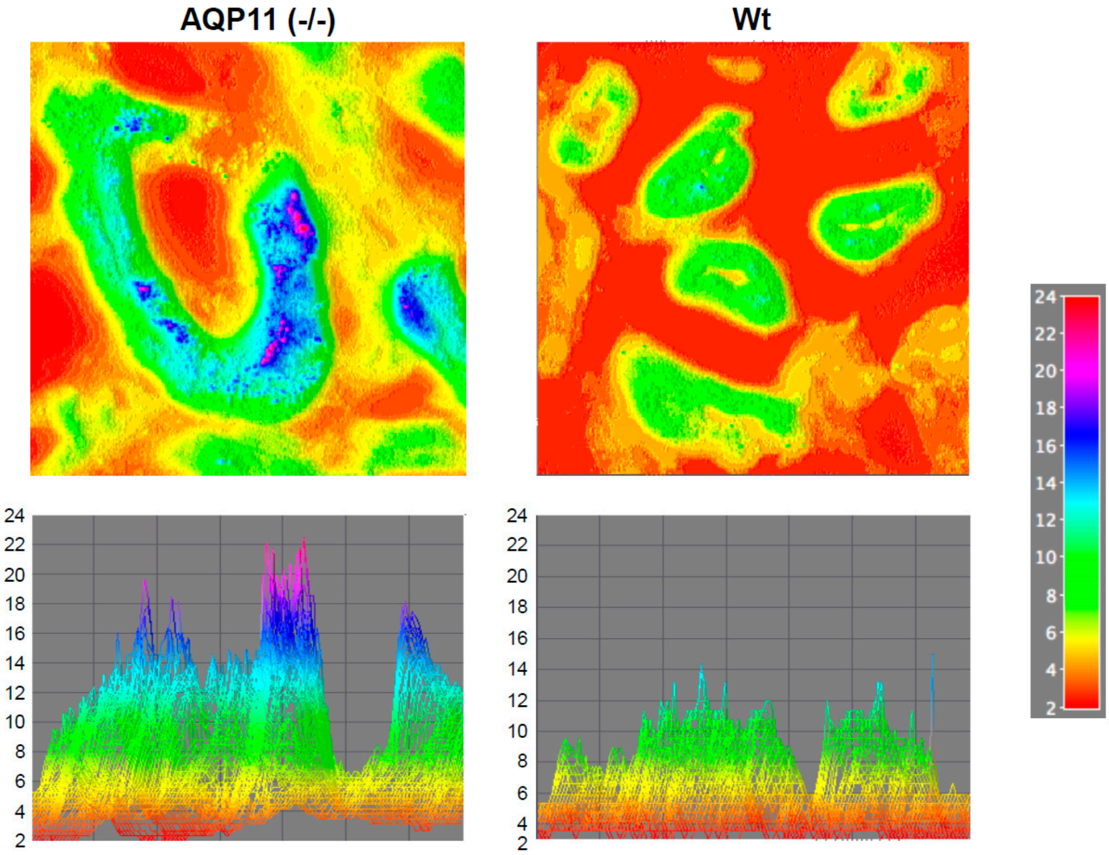


**Figure S3.** The GFP expression of puncta were analyzed by interac@ve 3D surface plot of Image J for the proximal tubule in Figure 3D,J. The intensity of fluorescence was shown as a heat map indicator on the right. The level of heat map under 12 indicates a background.
